# A multidimensional approach to analytical science—from invention and prototype to business on an international scale (1939–1976)

**DOI:** 10.1155/S1463924684000353

**Published:** 1984

**Authors:** R. Stanley

**Affiliations:** Department of History of Science and Technolooy University of Manchester Institute of Science and Technology PO Box 88 Manchester M60 1QD UK


Journal of Automatic Chemistry, Volume 6, Number 4 (October–December 1984), pages 175–185

### A multidimensional approach to analytical science—from invention and prototype to business on an international scale (1939–1976)

**R. Stanley**

*Department of History of Science and Technology, University of Manchester Institute of Science and Technology, PO Box 88, Manchester M60 1QD, UK*

### Introduction

Analytical techniques in chemistry have progressed from 'rule-of-thumb' methods practised in antiquity, through a period when micro, semi-micro, gravimetric and titrimetric methods etc. were extensively applied and were accepted as the classical and the standard way of analysing materials of pure research and industry. Improvements in the electronics field were applied to analytical methods which had to furnish more comprehensive, more accurate and faster results from more competitive industries. Such industries began to make more varied and sophisticated products of technological innovation following, in particular, the Second World War. This kind of progress has naturally paved the way for the automated equipment so common in today's laboratories. Though semi- and fully-automated processes have been applied over the past 30 or more years, automated methods have been generally available for about 20.

The Technicon AutoAnalyzer made its commercial dêbut in 1957, and judging by its varied analytical applications described in many scientific publications, it has had a dominating influence in the field of automatic analysis. The concepts of continuous flow with dialysis and then analysis, were both revolutionary and innovative, and, as such, this analytical set-up may be used as a reference landmark in the recent history of automated chemical analysis.

Many people regard Leonard T. Skeggs, Jr (see figure 1), inventor of the AutoAnalyzer, as the founder of automatic chemical analysis. Indeed it was from his paper in 1957 [1] that a distinct discipline emerged, now called 'automatic chemical analysis', or 'analytical science'. Invention is so often born of necessity, and it was from the large and increasing numbers of routine clinical analyses that were being demanded from laboratories in the USA in the late 1940s, that Skeggs thought that 'it would be damned nice of we had a machine to do this sort of thing' [2]. The Veterans Administration Hospital in Cleveland, Ohio, where he worked, epitomized a situation apparent throughout the country. The number of clinical laboratory analyses were then estimated at 35 M a month, with a projection that this figure would double during the following 10 years. Such a situation was exacerbated by the fact that technicians able to perform such work were becoming rare. Semi-automatic pipettes, photo-electric colorimeters and flame photometers only provided light relief. Besides, the doctor could not order all the laboratory work he wanted, it would have just cost too much [3]. Out of such a background, came an idea which was to evolve into the concept of continuous-flow analysis and marketed under the trade name of the AutoAnalyzer by the Technicon Instruments Company.

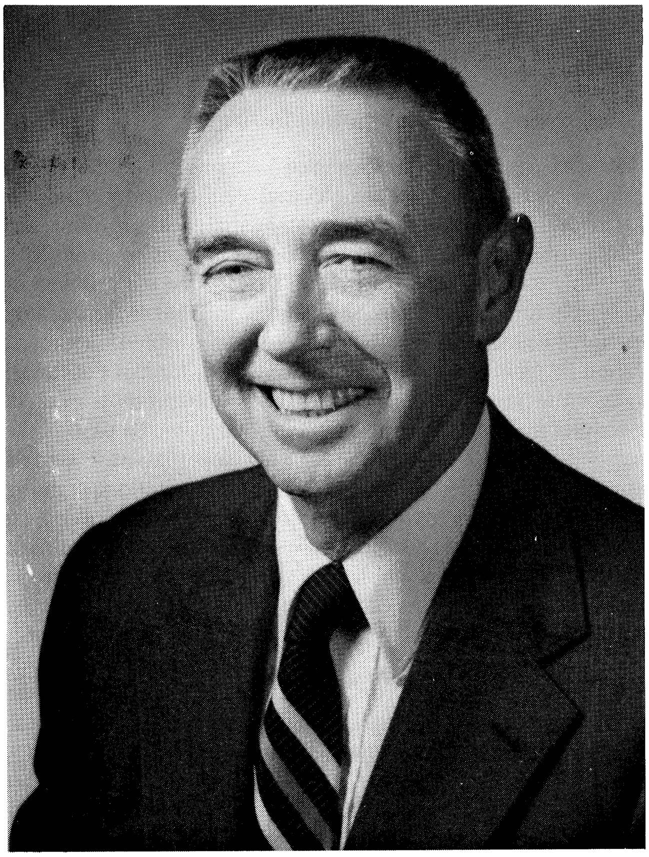

*Figure 1. Leonard T. Skeggs, Jr, Ph.D.*

### Skeggs and the artificial kidney

In 1943, Skeggs and Bernhart produced a paper which was concerned with determining the iron content of crystalline haemoglobin [4]—a long and tedious procedure lasting some 8 h or more. Skeggs later became involved with Jack Leonards and V. C. Myers in hypertension studies with respect to 'blood pressure determination in rat's tail'—1946 [5] and 'the effects of hypertrophy on the chemical composition of the rat's cardiac muscle'—1948 [6]. It was during these earlier years that both Skeggs and Leonards had invented an improved version of the artificial kidney—'a pretty hot item at the time, according to Dr Skeggs'. Prior the Skeggs's and Leonards's invention, much time and effort had been given by such as J. Abel [7] and L. G. Rowntree [8] in producing a substitute kidney [9–11]. As a substitute for the normal body functions, such man-made devices have allowed recovery of otherwise irreversibly damaged kidneys. Skeggs's and Leonards's approach to the subject had been originally drawn on Skeggs's kitchen-table. The idea was to replace the former cumbersome dialyser set-ups by a portable stack of 12 small dialysers (12 in × 18 in × $\frac{3}{16}$ in), each comprising two grooved rubber pads [12]—the first version [13] employed only one cellophane sheet. The patient's arterial blood containing urea was able to flow between the cellophane sheets, with the dialysing solution flowing in the counter current through grooves in the rubber pads. Blood then moved through the other dialyser units in series. It was then returned to the patient almost devoid of urea. The two pieces of cellophane were separated at either end by two identical pairs of steel plates. This type of artificial kidney serves as the basis for many models currently in use, although it has been replaced by improved designs following its considerable use during the 1950s. The dialyser became an important and versatile component of the AutoAnalyzer system, and it began to be used in a variety of filtering processes whereby interfering or unwanted materials could be removed from continuous-flow systems.

### Skeggs and the bubble machine

The idea began when Skeggs used the concepts of dialysis to analyse and screen blood samples and other biological fluids. In setting out to automate the extensive analytical procedures of clinical laboratories, he subsequently went on to produce a continuous-flow analytical train. He overcame the problem of getting a protein-free sample by dialysis, and that of maintaining sample integrity by air-bubble segregation. Formerly, standard blood chemistry analysis necessitated the addition of a chemical reagent to serum samples, and this would promote protein precipitation—a very time-consuming operation. By passing the mixture through a filter paper, only supernatant materials would be eluted. Skeggs's work was quite revolutionary in the field of continuous analysis. One innovation was that of using a multichannel peristaltic proportionating pump. With this a number of solutions could be pumped simultaneously at constant, but different, speeds. Thus, volumes of liquids could be automatically dispensed and mixed in fixed proportions determined by the appropriate choice of pump tubing with individually prescribed diameters. Such methodology replaced the more normal modes of reagent dispensing. By controlling flow rates instead of measuring volume, it became possible to establish a steadily recorded base-line condition on the recorder. So arranged, any sample and standard variations were highlighted upon the steady-state base-line. Perhaps the most profound technical innovation in Skeggs's system was the fact that small sections of the flowing analytical stream could be isolated by air-bubble segregation. These air-bubble barriers helped to prevent the mixing of following samples which flowed in the analytical train. Moreover, such segregation helped to scrub clean the inside walls of the tubing and assisted in the mixing of the separate, but close, liquid streams flowing through the horizontal glass coils incorporated in the flow path. The whole system was unique for its versatility—its success being derived from utilization of air bubbles.

### From prototypes to invention

As Skeggs was preoccupied with other intensive work during the day, his 'leisure' time was taken up in developing his instrument. His basement laboratory provided a convenient, but only too often cold, location in which to initiate work in a prototype in 1950. Often working on several nights in the week and until the early hours in the morning, by May 1951 an effective machine had been assembled [14].

Figure 2 illustrates this first machine. The assembly incorporated a Coleman Electric Colorimeter, as well as a two-grooved plastic plate dialyser clamped at the periphery with a piece of cellophane between. Some of these parts were enclosed in home-made timber structures. The colorimeter did not incorporate a recording mechanism, and therefore 'percent transmittance values' had to be recorded from the scale, written down and plotted on a graph from which urea nitrogen in blood samples could be calculated. 'It was pretty crude, but it worked'—said Skeggs [15]—and even the crude prototype was giving better results than the manual methods. Besides, it was obvious when the machine began to malfunction as the figures it produced were so spurious.

Figure 3 shows Machine No. 2 (November 1951) which featured refinements both structurally and in the components used—this time a chart recorder was incorporated. This system resembles the one patented in 1957. Skeggs's prototype, with its simple proportionating pump, mixing coils, dialyser and non-recording colorimeter adapted for flowing streams, was thus capable of eliminating the most frequent sources of error in clinical analysis: the mundane tasks of sampling and measuring chemical reagents and the accident-prone transfer operations involving the sample on its way to the colorimeter for final analysis. Skeggs's system and analytical philosophy were right for the time and welcomed by many when clinical analysts in the USA were being pressured for more determinations and for new methods of analysis to carry out what had previously been undetermined constituents—such demands being expected without an increase in budget or in the number of technicians performing the analyses. Skeggs had concentrated on glucose and blood urea nitrogen analysis, as these were the most common requested by physicians, and the first machine was capable of analysing for urea nitrogen in blood, having been designed to do so. A more detailed description of the operation of the early system using pre-heated coils for incubation at 55°C has previously been reported [16]. Not only was this modular approach to dialysis, proportional pumping and air segmentation unique, it offered flexibility in that a variety of flow patterns could be designed around a multitude of different analytical processes. Events have since illustrated the reality of this fact, evidenced by so many diverse applications of the system which have been published in different scientific journals.

### A chance visit

> But prototypes are only prototypes. It wasn't until a chance visit to Cleveland hospital by a Technicon salesman that Skeggs' dream became a reality [17].

Like so many inventions and discoveries throughout history, the events leading to the adoption of the concepts of the 'bubble machine' into clinical research and industry are complex. It was on a Friday morning in February 1954 that C. R. Roesch of Technicon was introduced by Dr Joseph Kahn to Leonard Skeggs and his 'interesting piece of junk'. This 'junk' formed part of the expanding research facilities of Crile Veterans Administration Hospital in Cleveland. About this time Skeggs was receiving acknowledgement for his work as co-founder of the redeveloped dialyser, and he was progressing in his studies on hypertension which is still his major preoccupation in life at the expense of instrument development. Roesch was quick to realize the potential of this pumping machine with its associated tubes everywhere. Skeggs was held in high esteem by local pathologists. A hurried telephone call to Technicon in New York received a more than enthusiastic reply to 'get Skeggs and his machine to come East no matter what'. (At this time Technicon were well-known as manufacturers of the Autotechnicon—a device for carrying out tissue biopsies.) Much persistence by

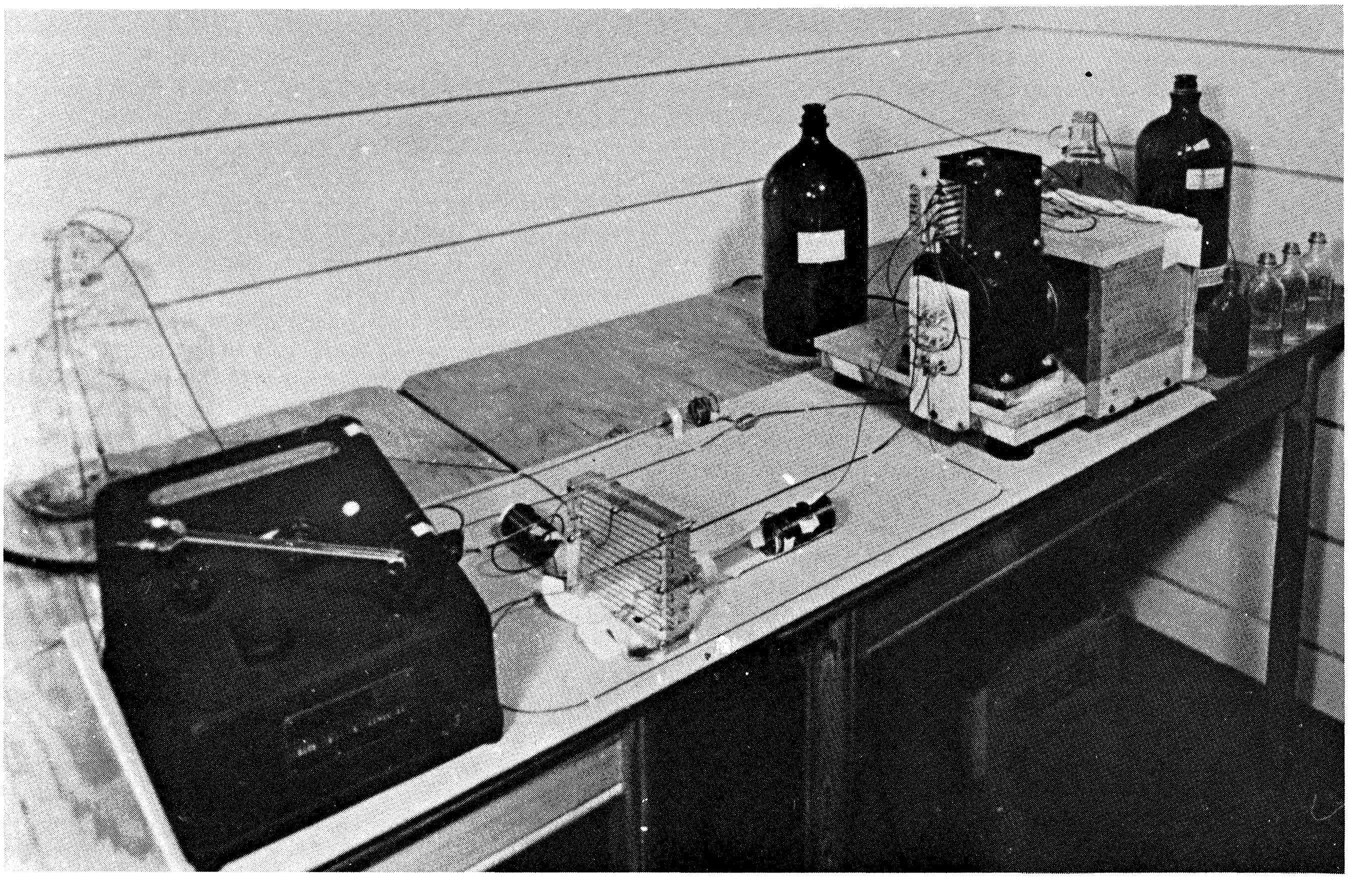

*Figure 2. Prototype No. 1 of the AutoAnalyzer (May 1951).*

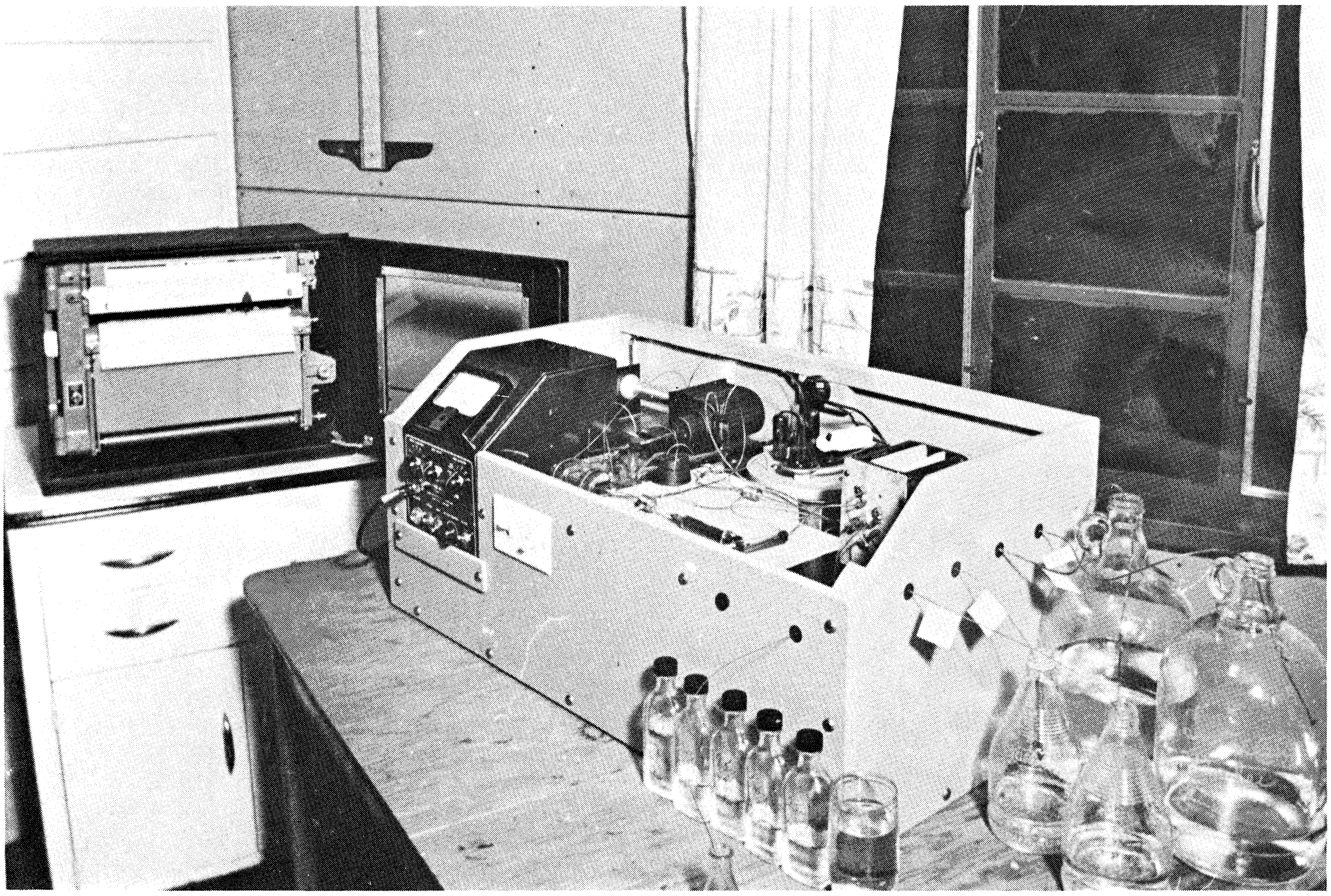

*Figure 3. Prototype No. 2 of the AutoAnalyzer (November 1951).*

Roesch pursuaded Skeggs to go. Technicon were extremely fortunate, for, unknown to Roesch, Skeggs actually had a contract in his pocket which he was ready to sign with a small company who were interested in his system. Other companies had actually turned him down—some saying to come back after securing a patent. Waiting for patents to materialize can take years, and such a myopic view by other interested parties ultimately led Technicon to capitalize on all Skeggs's inventions.

### Demonstration, adoption, contract and a patent

Skeggs and his wife loaded the prototype analyser into their car and drove to New York; in Technicon's own premises, they nervously demonstrated the system using freshly drawn samples of their own blood. The strip-chart recorder furnished a rapid analysis. Of course the Technicon people were convinced that the concepts involved in continuous analysis were revolutionary and that it had far-reaching implications and applications. The system was ingenious and practical and an on-the-spot decision was made 'to finance the development of that system, to take it from the theoretical to the operational stage' [18]. The Chairman of the Board of Technicon Corporation, Jack Whitehead, noted later that perhaps they would never have undertaken the venture if they would have understood the dimensions of the task and the size of the investment.

The prototype analyser was first built at a cost of about $1500 in the early 1950s. Very shortly after the demonstration, a contract was signed and Technicon vigorously researched and developed the prototype. In 1957, after four years' development at the New York research station, a commercial instrument, the 'AutoAnalyzer', was launched. (See figure 4.) Naturally enough, the system was just what clinical laboratories were crying for. The three original AutoAnalyzer methods measured levels of glucose, urea nitrogens, and calcium in human serum.

The first systems that were installed at the National Institutes of Health, and Cook County Hospital, Chicago, demonstrated the speed, accuracy and reliability of the AutoAnalyzer in performing those tests. At the same time, new tests were being developed by Technicon, and within a matter of months, several other clinical determinations were being performed on the AutoAnalyzer. Some indication of its immediate success can be gauged from the fact that 50 units were sold in the first year, 4000 by 1963, and 18 000 by the end of 1969. US Patent number 2,797,149—'Methods of and Apparatus for Analysing Liquids Containing Crystalloid and non-Crystalloid Constituents'—became effective on 25 June 1957, application having been received by the United States Patent Office on 8 January 1953 [19]. The patent contained 26 detailed claims protecting the invention, including the following statement: 'It is intended that the patent shall cover, by summarization in appended claims, all features of patentable novelty that reside in the invention' [20]. Details of the objectives of the instrument appear in the text of the patent, together with illustrations of instrumental details and flow-diagrams which indicate the potential multiplicities of flow circuits possible, and of chemistries applicable to the system. The success of the Technicon AutoAnalyzer relied on the strength of the patent as events were to show. A well-known company had copied the AutoAnalyzer system [21 and 22]. They were taken to court and were unsuccessful in the case. In more recent years, John Whitehead [23], Senior Vice President, Research and Development, Technicon Corporation and a descendant of the original founder of the company, shared the same business philosophy: 'If you want to be a pioneer in the development of analytical instrumentation it's a risky and expensive business... If people can come in and make a copy of your work at little expense, they may get all the benefits and you have taken all the risks'.

With strong patents, the company could freely display its wares and fully exploit the instrument's market potential. It has been very apparent that Technicon's AutoAnalyzer patent was also respected by instrument users in Britain. The patent certainly protected the interests of Technicon, but in some circumstances, it may well have restricted further developments in particular fields of chemical analysis.

Skeggs published his often-quoted landmark paper—'An automatic method for colorimetric analysis'—in September 1957 [24], indicating that the method eliminated the need for step-wise measurement, manual addition and processing of samples and reagents, and removal of blood proteins by classical methods—this being performed by continuous dialysis. The way in which the commercially developed system functioned in its

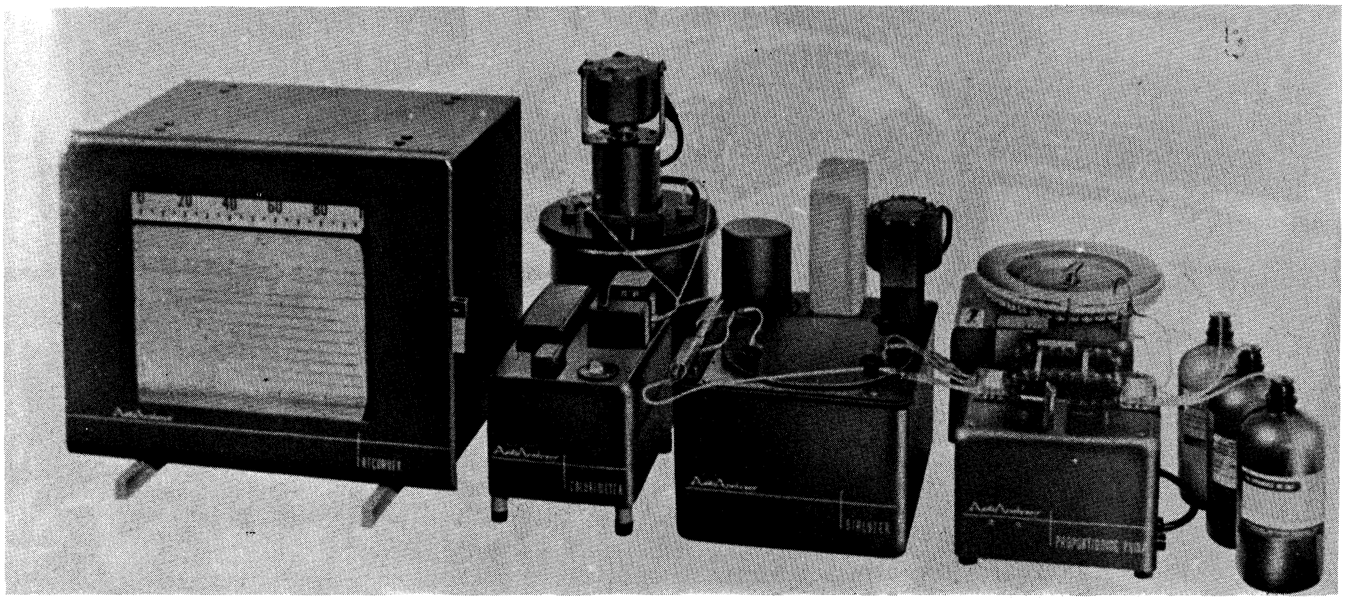

*Figure 4. Technicon AutoAnalyzer AAI—commercial instrument (1957).*

normal mode has been well tried and tested and need not be detailed here, save to list the essential features of such a modularized set-up: rotating and aspirating sampler; peristaltic proportionating pump and manifold; thermostatically controlled heating bath; colorimeter; and automatic recorder. The beauty of such a modularized system was that different analytical chemistries could be adapted in a vast number of configurations. What did the system really have to offer? It did not take long for scientists other than clinicians to realize the advantages of analytical flexibility, accuracy, reproducibility, reliability, automation, labour saving, continuous analysis of large work-loads, verification of calculations, relief of manual fatigue, a possibility of increasing work-loads, provision of extra analytical service, a decrease in glassware, and a reduction in cost per analysis (when the number of analyses required is greater than about five or 10 per day). Since the time when Skeggs presented his paper in 1956, the procedures and concepts opened up a new field in laboratory techniques. 'Since this presentation, automation in clinical and analytical laboratories has grown rapidly', and, according to Andrés Ferrari in 1962 (he was then Director of Research, Technicon Instruments Corporation, Chauncy) it had 'become firmly entrenched' [25]. Ferrari's paper demonstrates the tremendous impact that the AutoAnalyzer made. Indeed, Ferrari's article intimated at further revolutionary development by Skeggs and H. Hochstrasser [26]—a system capable of performing sequential multiple analysis (SMA). This system was a natural progression from the AutoAnalyzer and it could perform a series of analyses simultaneously for every patient selected by the attending physician and pathologist. Such a system could provide a physiological profile of a patient so that the physician could base his final diagnosis on clinical and laboratory findings. The development of this system has had far-reaching implications and consequences in other branches of analytical science, water management for example. The type of profile that the system was capable of charting could in some cases highlight otherwise undetectable deficiencies in a patient's health. So the system was, of course, patented.

Leonard T. Skeggs was to be involved with the Technicon Company in taking out a series of patents over a number of years [27], and Technicon were skilled in the art of applying for them [28].

### The life work of Leonard T. Skeggs, Jr

The life of this man to date is both fascinating and distinguished [29 and 30]. For his development work on the AutoAnalyzer, he received the Fleming Award (1957), the Van Slyke Medal (1963), the Ames Award (1966) and the American Chemical Society Award (1966). There were also many material advantages. Through indirect influence of the famous Harry Goldblatt, and the influence of Dr Kahn who had worked with Goldblatt, Skeggs was led into studies on hypertension. This complex research still occupies a great deal of his energies involving biological substances such as renin, angiotensin I, II, and III, proteins, amino-acids, vasoconstrictors, specific enzymes, blood plasma, pseudo-renin etc. 'This hypertension business is the hardest problem I've ever worked on. I just don't have the time for instrument work' says Leonard Skeggs, much to Technicon's disappointment. In their paper, in 1975 [31] and 1976 [32], Skeggs *et al.* give many references to the work accomplished with the renin-angiotensin systems and elevated blood-pressure in various animals such as dogs, rabbits and rats. The same authors published another paper in 1976 [33] on hypertension, and the complications of this biological system are only too apparent in the introduction. Since the beginning of 1948, Skeggs has been actively involved in this kind of work. Of retirement he said: 'Retire? You don't retire, you're supposed to work, aren't you? There's no time for retirement'. He attacks all problems with the idea that there is a better way to solve them than the obvious. Humbly attributing personal success to other people's influences, up to 1978, he had received many accolades, including the American Purple Heart, academic successes, board membership, elected member of scientific organizations, and had 79 scientific publications and owned 20 American patents [34]. A more comprehensive account of the life and work of Leonard Tucker Skeggs, Jr. is given in the book *Riding Along*, which was written by a person who Mr Skeggs has referred to as a 'very biassed individual'—his wife [29].

Leonard Skeggs has had a fruitful career through scientific discipline and dedication, and it may be fair to say that it has been through his intuitive exploits that the Technicon Corporation have been able to capitalize on his talents and grow into a formidable and international company with all-embracing back-up services and professional personnel. Such a multidisciplinary approach to a systems problem is inherent in the AutoAnalyzer set-up, which interfaces distinct features of analytical processes from sampling through to presentation of analytical results.

### Technicon—from humble beginnings to an international organization

As a preamble, it must be said that this overview of one company's successes is not meant to boost Technicon products, but hopefully will serve as a model illustrating where business skill has been combined with technological innovation. Much philosophy, so often hard learnt, formulated Technicon's operations in national and international markets. Contemporary companies, including some in the UK, could well copy such strategies. In the context of their operations, Technicon have looked at a full spectrum of aims and objectives for both short- and long-term enterprises: 'a multidimensional approach to a multifaceted problem'.

In the early 1920s, a company was founded in New York and its first products were thermometers and laboratory glassware. In 1930, the owner, E. C. Weiskopf, built an automated machine for tissue processing: this was the Autotechnicon. Both machine and inventor were rejected by leading American pathologists, and it was not until the Second World War that tissue processing by machine became a reality. The shortage of manpower in pathological laboratories encouraged the installation of this advanced machine, which could accomplish, overnight, work requiring three to four days of classical procedures. Modest beginnings were followed by new inventions, such as 'Lab Aid'—a unique system for filing laboratory slides. After E. C. Whitehead joined his father's operations, an ambitious research and development programme produced new and significant products. These instruments included a portable micro-projector, the 'Scorpion', which for the first time used a mercury arc as the light source, and the first fraction collector in the World designed in conjunction with Nobel prize-winners Drs Moore and Stein of the Rockefeller Institute. Quickly following these products, the 'Technicon Huxley Respirator' appeared in the early 1950s, this being the first chest-abdomen portable respirator for polio patients. The machine's unconventional concepts freed the now mobile patient from internment in an iron lung, and it became largely redundant following the introduction of the Salk vaccine. Other products included a three-channel direct-writing 'cardiograph', and a microscope lamp which could illuminate any microscope. Advanced models of the Autotechnicon were developed and are still working in pathology laboratories. Technicon continuously grew by developing only new and unique equipment—never an improvement on a product already on the market. Since the time that the

AutoAnalyzer was launched in 1957, the magnitude of Technicon's operations have grown exponentially.

With headquarters in Tarrytown in New York, Technicon have a world-wide staff of over 4000 employees located in 48 manufacturing, sales, services and distribution centres in 28 countries. In its field, it is one of the largest and most multinational of organizations. By adopting hundreds of AutoAnalyzer tests, entry into vast industrial fields as well as clinical fields became feasible. Technicon operations encompass over 2000 hospitals with over 200 beds, and 500 large private laboratories. There are about double these numbers in Europe, Asia and the Far East. With an estimated 15% per annum increased testing in these establishments—a figure which is more or less consistent with figures quoted in the UK in the 1930s of 15% [35], and from the 1950s onwards of 20% [36]—the development of clinical analysis and the demands created for increased testing is well documented to modern times [37]. Over the years, Technicon's product volume output has grown fairly consistently at about this steady-state condition. Consistent with such expansion, 'Technicon Medical Information Systems' (TMIS) is a very modern merchandise marketed by the company aimed at approximately 2000 beds in the USA. They found that 30% of their soaring costs— estimated at $40 billion for 1976—could be attributed to information handling, and such a system was established to cope with this and other tasks.

The success of the company is based on firm business philosophy and knowledge of such disciplines as physics, mathematics, electronics technology, management science and technology. Linked to such operational principles was a policy of disseminating knowledge through international symposia, and the subsequent publication of the proceedings. By publishing technical papers and similar material on continuous-flow applications, Technicon were able to pass on knowledge to be shared by all analysts. This facility was augmented by the company's teaching programmes where users of the equipment could be instructed in the principles, maintenance and potential of Technicon products—this free training-course being available to all customers. With thoroughly reliable and professional after-sales services and extensive research and development programmes, Technicon's fortunes are perhaps an example for many other organizations to follow, and perhaps a study of this organization's approach may indicate where others have failed, particularly in Britain. P. B. Stockwell's definition: 'A multi-faceted and multidimensional problem the various aspects of which are fully interlocking a multidisciplinary problem' [38], certainly seems to epitomize a purpose that Technicon had adopted many years previously by getting the right people at the right time and protecting the resulting interests with a strong patent. The company and its objectives have been presented over the years, and certain key products are seen by the company [39] as definitive landmarks in the history of Technicon: the Autotechnicon (1939), the AutoAnalyzer (1957), the SMA computer-linked system—SMAC (1972), and TMIS (mid 1970s). So exactly what business philosophies really contributed to such outstanding economic and technical success?

### Technicon—the formative years

The record shows that the business of the Technicon Corporation was operating under that name from about 1939 in a loft in the Bronx, New York City (149th Street and Park Avenue). This company took over from the Empire Laboratory Supply Co. established by Edwin C. Weiskopf (1892–1968) in 1921. The company dates back to before the turn of the 19th century to the experiments of Abraham Weiskopf, the grandfather of Chairman E. C. Whitehead. By placing some nitrogen gas on top of mercury in his glass apparatus, he had invented a new type of thermometer that could be used by industry. This provided sufficient capital for him to be able to give up his job as a Chicago district saleman with a Milwaukee brewing company, and establish his own firm. Abraham Weiskopf liked to experiment in his spare time and had previously come up with other inventions, A US embargo during the First World War on contemporary German products (such as thermometers) opened up new business opportunities, and Abraham decided that his son Edwin should join him in the company, although at the time Edwin intended to pursue his future in merchandizing. Nevertheless, the idea of experimenting with merchandizable products seems to have been set as a precedent at such an early stage in the embryonic business. Edwin Weiskopf had graduated from Chicago University and was a contemporary of Lessing Rosenwald, the son of Julius Rosenwald. (Julius was the son of a Westphalian pedlar who had turned Sears Roebuck into the world's biggest general store.) Edwin had commenced working with Sears as an assistant buyer and, when approached by his father, Edwin explained that he was 'gaining an invaluable education fron the great Julius Rosenwald himself'. Abraham arranged to have his son sacked by Sears and recruited to the family business. Edwin's experience with Sears was to prove invaluable in the subsequent growth of the Technicon Corporation. Abraham always expected obedience from his son, which, fortunately for the company, did not always materialize, for example when, in 1916, E. I. du Pont de Nemours called for tenders for the supply of thermometers. Thinking he could not meet the huge deliveries that were required, Abraham threw away du Pont's letter. However, Edwin decided the order must be secured, recovered the letter and went ahead on his own initiative, even though he could not deliver the full order at once. Edwin's actions were consistent with his training at Sears, where Rosenwald taught him that a company did not necessarily have to hold stock prior to receiving an order to sustain corporate growth, and that more often than not it was perhaps more prudent (for the company) to use a supplier's capital to purchase materials after the customer has shown interest in placing an order. This approach was used to raise the money for the development of the first Autotechnicon system in the 1930s. Paying suppliers promptly was another policy favoured by Rosenwald, who apparently believed that everyone benefited—the company, the supplier and the customer. Edwin later recalled that his father disliked unpaid accounts, and the firm never faced liquidity problems, even during the depression years of the 1930s. He always insisted on paying suppliers within a matter of three days. Abraham made a show of outrage over the thermometer incident, but was secretly delighted, and his son remembered: 'He only told me years later how pleased he was—just after I had repeated my earlier success by contacting another major company after he threw away the letter'. Very little money was ever borrowed, although Edwin had taken out a $5000 personal loan to start his business in 1939. Much more credit could have been obtained during the early years, but he held fast to the principles of rapayment within three days. However, realizing that credit had become an intrinsic part of most business arrangements, he eventually made the unprecedented move of borrowing money from the bank—just to establish credit facilities, Technicon did not borrow money again or sell equity until December 1969 when the company made 1 M public shares available on Wall Street. Sales then exceeded $78 M. The healthy state of the company in 1969 was described by Edwin C. Whitehead in his opening remarks to the stockbrokers in Technicon's 1969 *Annual Report* [40] when several new and patented instrument systems were introduced [41].

### Father and son part company

Weiskopf's thermometer trade began to dwindle in post-War America due to inflation and renewal of cheaper German imports, and they ceased manufacture in 1921. It was at that time that father and son parted company, Edwin C. Weiskopf established his Empire Laboratory Supply Company and set out to build a modest, but flourishing, import agency. He still used the Rosenwald principle—'sell first, buy later'—to build up his business. In 1929 he met two pathologists—Harry Cross and Harry Goldblatt [42] 'who would change his life—and the nature of laboratory analysis—forever'. They had devised an innovative automated system which could replace the tedious methods involved in preparing human tissue for diagnosis. Formerly, these often irregular tissue diagnoses could take up to four days of work. Weiskopf soon gave this new and revolutionary instrument the flamboyant name of the Autotechnicon, to which the author has already referred. Such machines in 'mono' and 'duo' form have been used as clinical laboratory workhorses since that time. The Technicon Company, under a licensing agreement with Cross and Goldblatt, made and sold only six Autotechnicons between 1929 and 1939 for about $250 per unit. The constraints of the depression hardly gave incentive to the sales of such machines which were designed to eliminate hand labour.

Edwin C. Whitehead (son of Edwin C. Weiskopf and Jack as he was known to his friends) was appointed shipping clerk to the infant company in 1939. The events of the Second World War and the associated shortage of trained laboratory technicians and pathologists sparked off more interest in the Autotechnicon system. In an age of poor economic growth, Edwin the elder had secured sales of this instrument to hospitals, the managers of which believed this machine to be 'unscientific'. The dynamic influence and personality that the elder Edwin forced upon his potential customers was only too apparent. He secured a sale to the New England Hospital, where one pathologist was most apologetic for keeping him waiting for the comparative results from the test machine [43]. Jack Whitehead became a door-to-door vacuum-cleaner salesman after leaving Virginia University. The appointment with his father's firm as a shipping clerk eventually followed, and he promoted himself to the position of Technicon's 'first-and-only saleman'. During his early career with the company, the young Whitehead sold 100 Autotechnicons when, during a similar period, only 25 were sold by six other salesmen from another company anxious to take on the distribution of the system. This admirably demonstrated Jack's capacity for marketing Technicon's merchandise. His poor eyesight prevented him being drafted into the armed forces—besides it was considered more important for him to promote sales of the Autotechnicon for its usefulness in the War effort. By 1943 over 200 units were in operation. Profits from these allowed the company to develop other sides of the business. Various publications have described the earlier [44] and later models [45–47] of the system.

### Prototypes and inventions—some early lessons

The Technicon company were taught an early lesson in business during its formative years which would serve them well in the future. Some new products did not survive long, whilst others were the prototypes of the ultra-modern and sophisticated instruments of today. Some were introduced at a time when industry did not indicate demand. Many years were involved in the development of the first direct-reading electrocardiograph. Realizing that three-lead electrocardiography was about to supersede the single type, they developed such a system only to be told that vector cardiography was then the latest technology. Back to the drawing-board, a three-lead vector cardiography unit was produced by Technicon. It used recording paper and was linked to an oscilloscope screen. Ironically, Technicon marketed this system for $3500, only to find that a contemporary company developed precisely the kind of unit originally produced by Technicon, and this was sold for a mere $700. Such experiences as this again were to mould company philosophy. Of course, strong patents prevented this kind of setback from happening again.

The company went on to develop a whole range of new and innovatory instruments [48] and appeared to adopt a policy that closely followed the maxim: 'if at first you do not succeed, don't try again'—that is, a company should never try to improve on a product that is already on the market. By developing only new and unique equipment, the company could ensure that they would have a continuous growth pattern in the future.

### Tackling professional secrecy by book writing

Scientific secrecy abounded during the Second World War and the early post-War years, and in the medical profession the situation was similar, such that it was discovered that there existed a lack of uniformity in the use of the Autotechnicon. Worse still (for Technicon) there was a lack of documented case-histories concerning its usefulness, largely because pathologists were not over-anxious to share their experiences. Naturally such a situation was counter-productive to an effective and coherent strategy for marketing the instrument. Technicon sent detailed questionnaires to all its customers asking them about the use of the Autotechnicon; more than 300 replied, furnishing the company with enough information for a book in histological methods. This publication became the cornerstone of Technicon's marketing strategy which has served them so well.

### An early lesson in patents

In the late 1940s, W. H. Stein and S. Moore (1972 Nobel Laureates for work on ion exchange) evolved a prototype for a fraction collector [49], which was vital for carrying out automatic amino-acid analysis. After Mikhail Tsvett's classic paper dealing with chromatographic separation of amino-acid from a protein-laden mixture and using a potato-starch-packed tube, such techniques lay dormant until work by Martin and Synge in the early 1940s [50]. In 1949, W. E. Cohn at Oak Ridge National Laboratory, USA, used the Tsvett concepts of adsorption and absorbed the hydrolysis products of nucleic acids on columns of cation- and anion-exchange resins, and stripped them off individually by passing hydrochloric acid or buffer solutions. The original analysis could run into days. After Moore had approached Technicon about the automatic fraction collector, an intensive programme of development led to such a device being readily available. It allowed the science of chromatography to advance as a research tool. Technicon had promoted the fraction collector business, though at the Rockefeller Institute, where Stein and Moore carried out their research, it was the policy not to patent inventions. As a result, many other concerns quickly began selling fraction collectors. Technicon gradually withdrew from the business, though still selling versions of the fraction collector in the mid 1970s [51]. This experience taught the company an important lesson about having strong patents—this practice put an end to 'surprises from competitors that were a daily occurrence in the early days of the fraction collector experience' [52]. An extensive list of Technicon-owned patents between 1962 and 1976 [53] highlights the kind of exponential growth pattern apparent over that period.

### Book writing and the concept of the symposium

The head of Technicon's research laboratory—Andrés Ferrari—and Jack Whitehead, decided to write a concise set of instructions to be supplied with each AutoAnalyzer to ensure correct commissioning of the system. This was to be followed by a call from a Technicon service person. It soon became apparent that, because the new AutoAnalyzer concepts were so complicated in application and novel from the outset, writing such a book would be a most overwhelming task for them. It was therefore decided to teach the first 10 customers about the principles involved in using such a set-up, as this might indicate what should be included in any manual. This exercise still did not enable them to write an instruction manual and 50 people were invited—soon their teaching strategy became an outstanding success and a permanent feature of the company's marketing practice. Eventually a training school was established in a 25 000 square foot network within Tarrytown's Technicon Science Centre, incorporating a demonstration laboratory, classrooms and a 300-seat lecture-theatre. The training school idea was copied in other parts of the USA and overseas, such as at the Basingstoke site in the UK. Each branch carries out its own training in the language of the country where it is situated: at Basingstoke, for instance there is a throughput of about 300 students per year with Turkish and Farsi as language options. By 1970, 12 years after the inception of the education programme, more than 19 000 students had been awarded diplomas by the Customer Education Department. Another spin-off effect for Technicon was that the American Commission on Continuing Education of the American Society of Clinical Pathologists produced a film—*Continuous-Flow Analysis* (1969)—which gave the story of automation in the chemical laboratory. The motion-picture was awarded a silver medal at the New York Film Festival as the year's outstanding medical film. A further side-effect was that, by 1970, courses in automated analysis instituted by Technicon, were included in a growing number of university curricula. In 1969, an instructor's course for educators was started at Tarrytown. In line with these educational philosophies, Technicon initiated the concept of the 'International Symposia on Automation'. Education and communication have proved to be the prime vehicles for disseminating information about automated analysis to academics and other interested parties.

New applications in the art of continuous-flow methodology meant that much diverse literature on the subject appeared, and Technicon considered that the scientists who were adapting AutoAnalysis to their own specific requirements could best disseminate their own specialized knowledge. So the company launched a series of symposia to allow user-physicians, researchers and technologists from various disciplines to share their ideas, experiences, and problem-solving. Such interdisciplinary cross-fertilization provided a valuable source of new methods, techniques and applications of automated analyses. The symposia began as informal gatherings in London and Paris in 1962, and matured into internationally respected scientific meetings. The first American symposium was held in New York City in 1964, and the next year's domestic and international meetings attracted thousands of delegates—many of whom requested reprints of papers. Like previous experiences with the Autotechnicon literature, it was soon realized that such scientific papers, once collated, would form the cornerstone of definitive literature of a new scientific discipline. Multilingual editions of *Automation in Analytical Chemistry* were published in 1965, 1966 and 1967. Technicon organized the publication of many other editions of these detailed compilations. Created by the late James Evans (who died in 1973—the first Managing Director of Technicon Instruments Ltd in the UK) the international congress idea has paid many dividends for the company, attracting world experts from such disciplines as clinical biochemistry, histology, animal studies, agronomy, environmental sciences, pharmaceuticals and biochemical profiling, computer science, nutritional science, and others from the many facets of industry. Their international symposia, which have always attracted impressive groups of speakers, are held in lavish settings around the world. The congress gatherings provide Technicon with the rare opportunity to introduce its new products to the world scientific community. The company never announces a new commodity until a range of new products has been put together, and then unveils them at the symposia. The international symposia nearly did not get off the ground—literally—because of a near crash in a shaky Second World War Bristol bomber chartered from a private company based at Gatwick Airport [54]. Nevertheless, this concept of disseminating knowledge, which has been outlined in more detail [55], again has been written into company philosophy.

### Research and development

According to John Whitehead (Vice President of Research and Development at Technicon and son of Edwin C. Whitehead), there has never been a 'me-too' product: merchandise is always produced in house, or by outside agencies, and perfected by Technicon, and then safeguarded with strong patents. As innovators, the company perceives that it is only as good as its last product and that it should foster expansion by research and development in the specialized field of analytical science and instrumentation.

During 1969, Technicon spent $4 879 000 on development [56], compared with $2 912 000 the previous year. Growth in research and development costs between the years 1965–1969 has been published as a histogram [57]. This highlights a commitment to expansion in R & D in order to maintain a leadership in meeting the requirements of automation in the industrial field. In 1971, $10·6 M was spent with the intention of expanding R & D in order to promote associate growth [58]. This represented a three-fold increase in this expenditure since 1968. Technicon's philosophy has been to shape its own future through research and development and the company has always dedicated its efforts to a 10-year time span to cater for a continuing flow of new products. A three-to-five year development programme is aimed at bringing basic research advances to the product definition stage. Shorter, one-to-three year programmes are set to extend the application of established methodologies in the clinical and industrial areas. Various specialized instruments are developed during the shorter programme. 'International task forces' were created to extend systems' versatility. A Programme of Grants for Research in Biomedical and Industrial Instrumentation was announced in 1974. Initially, it was perceived that those receiving such a grant could be awarded up to $100 000 per year for three years to develop proposals of outstanding potential. Each application was to be judged according to scientific value and viability in the future market-place. Invitations for grant applications to develop unique concepts leading to inventions of processes or devices were offered to qualified faculty members and scientists from colleges, universities, medical centres and health research institutes. This scheme for basic and applied research attracted prominent scientists and engineers with wide and vigorous interests in clinical medicine instrumentation.

Because Technicon always considered confirmation of scientific results as an essential part of the research and development process, it was a simple matter for them to prepare all the documentation on their medical instrumentation which was required by the new (1974) regulations of the Food and Drug Administration (FDA) concerning *in vitro* diagnostic products. Such information covered such issues as truth labelling, proof of claims and good manufacturing practice. Discoveries on the various parameters of government legislation and its impact on the laboratory were given in 1976 [59]. The company's attitude to research and development is that it is an investment in their future and not just an expense—the overall objective being the practical application of theoretical sciences to develop innovative products and services. Perhaps the most ambitious programme has been the more recent Total Medical Informations Systems designed to surmount some of the daunting barriers to hospital efficiency and productivity [60].

### Ancillary services—the total systems approach

Partly through necessity, because the company was involved in medical operations, Technicon products not only had to meet stringent operational requirements, but they also had to be reliable. Such demands were enforced by operating a strong after-sales service. Such a 'Total System Support' philosophy was nurtured in the early 1950s through involvement with the lightweight portable respirator for polio victims—Technicon established a 24 h back-up duty each and every day. Their equipment had to have built-in reliability, because, if a respirator was out of service for any length of time, it could quite simply cost a patient his life. Such systems had to be designed incorporating a support facility supplemented by a service network for replacement units with technicians available by telephone all the time. Of course, the development of the polio vaccine in 1954 virtually ended Technicon's respirator business. However, these experiences had moulded the company's policy on after-sales support. These inherited experiences were very apparent in 1963 in Technicon's operations in the UK [61] and this reliability became well-recognized [62].

The philosophy of the systems approach to automation in analytical chemistry is manifest in the capability of Technicon instruments. That philosophy has obviously worked, particularly in industry, as 1975 provided revenues of $18 690 000. This equalled 9% of total corporate revenues [63].

Technicon's all-embracing business philosophies did not end there. In 1966, they entered the chemical industry market with an assortment of chemicals and other consumables—some 500 types—for application around the world. Between 1970 and 1975, there had been more than a 200% growth in this business. In 1975, these sales amounted to $45 188 000—an increase of about 22% over the previous year. These revenues continued to grow, and in 1978 they were $73 M world-wide—this figure did not include materials consumed in Technicon's equipment on metered rentals. Individual systems had individual requirements, and to meet such a demand the industrial division of the company organized a special sales unit for each major market. Distribution centres assure prompt delivery of precise quality reagents that can be used uniformly and accurately on all Technicon's instruments. As well as modern chemical plants in Tarrytown (New York), Santa Ana (California), and Tournai (Belgium), distribution centres were set up in the US, Canada and Europe. In March 1970, the company established two manufacturing subsidiaries in Peurto Rico to produce electronic components, assemble instruments and make laboratory glassware to be used in those systems for the domestic and expanding Latin American markets. The UK manufacturing facilities were set up in 1958 at Chertsey. A completely integrated 120 000 square foot production plant was established at Swords (Eire) and it became the international servicing centre and technical training centre for Technicon personnel overseas. The Chertsey headquarters subsequently moved to new premises at Basingstoke, Hampshire. Providing services to 28 different countries with a population of about 317 M, this branch covers an area of operation of some 4·7 M square miles, and itself provides research and development, servicing, spares and back-up services etc. for the many analytical disciplines connected with Technicon. World-wide marketing had been established in 16 overseas offices by 1970, with headquarters in Geneva and agent representation in 64 other countries. This kind of expansion continued in subsequent years to such other countries as Japan, Korea, Taiwan, Hong Kong, Italy, Australia and so on. By 1971, the company had expanded its field force of engineers by 20% to assure total support for all their existing and projected placements.

Technicon's achievements in this field of back-up services are many and too prolific to mention. Suffice it to say that Technicon's world distribution centres and service outlets allow for prompt delivery of reagents, consumable supplies and parts; their branch offices serve simultaneously as service, sales and distribution outlets; and the major sales offices provide for customer training facilities. Complementing the service engineers, in the early 1970s, the company maintained technical specialists to follow-up on new customer's installations and assure optimum systems operation, give customer training in their own laboratories when necessary, and direct workshops and seminars.

The Technical Division set up an International Advisory Committee on Methods and Standards to assist the company in maintaining the advantages brought to clinical chemistry by automation. During 1973, a Methods and Standards Research Laboratory was opened in London to develop new or improved methods and standards for a range of biochemical techniques.

The company has been actively involved in international markets for over 20 years and these global interests, Technicon believe, penetrate the many complexities of world marketing, and have accelerated Technicon's world-wide accomplishments. Despite the prospective world-wide recessionary trend at the beginning of 1974 due to the escalating oil crisis, international sales increased by over 25% over 1973, with further penetration into established markets. It was a year when new markets were beginning to be tapped and preliminary contacts were made with the People's Republic of China [64]. Another major part of the company's supporting operation has been the Technicon Leasing Division. Leasing and rentals of analytical systems for 1975 representing 19% of the total revenues. Of course such success has continued.

### An overview of success

Many early lessons have helped to formulate company philosophy over the years, and to establish the company as an authority on the use and application of its own problem-solving equipment. Coupling this to original designing, construction and marketing talents, the company's advanced instrumentation led naturally to the concept of providing comprehensive systems dealing with all parameters of variability—i.e. the total systems approach to a 'multifaceted and multidimensional problem...'—an idea that Stockwell and co-workers are continuously trying to advance in the area of automatic chemical analysis in the UK [65]. Consistent with this all embracing approach to the interdisciplinary nature of instrumental analysis, and the automation of these techniques, Technicon in 1957 presented a total systems approach to clinical laboratories by way of the innovatory AutoAnalyzer, invented by Leonard T. Skeggs Jr. The impact that the machine had can be gained by the thousands of varied applications that have been forthcoming since its initial introduction into the analysis field [66], whether clinical, agricultural or industrial. Developments in analysis within the clinical field are described by L. T. Skeggs in 1969 [67], and E. C. Whitehead *et al.* in 1976 [68].

Everywhere, researchers in industrial laboratories began applying AutoAnalyzer techniques to their wet chemistries and technicians began tailoring continuous-flow methodology to their own specific requirements: content uniformity in the drugs industry; nutrient content in agriculture and so forth. Being a very portable system, it could be used in mobile laboratories to monitor municipal water-supplies for instance. Many areas of the industrial world began utilizing this instrument system, from metal works to textiles, to paper and pulp industries, to brewing, to ice-cream manufacture—the list seems inexhaustive. In 1964, the company went on to develop and market the sequential multiple analysis system (SMA) through the exploits of Skeggs and Hochstrasser [26]. In 1967, the company introduced the first SMA 12/60 system [69] which could automatically analyse for 12 tests of the laboratory's choice. The results it produced were not only individual chemistry test results, but a multiparameter analysis of a patient's condition charted on a graph for easy reference—a patient profile that could identify otherwise non-detectable diseases in a patient [70]. An evaluation of its performance was given by Finley *et al.* in 1969 [71]. Other configurations of SMA have followed such as the SMAC—'C' for computerized—and the second-generation SMA IIs (1976). The SMA system contributed in no small way to the substantial jumps in revenues realized by Technicon in over a decade.

In 1957 Technicon headquarters were based at Ardsley, New York, and the company was reaping the benefits of success by expansion. AutoAnalyzer sales had accrued $18·4 M in 1964. By 1967, the company employed hundreds of employees and turnover was just over $44 M per annum. In 1969, Technicon had realized $78·7 M worth of AutoAnalyzer sales. By 1972, the headquarters were located in the Technicon Science Centre, Tarrytown, New York, with an accommodation of nearly 90 acres. Income was in excess of $109 M [73], which was almost triple the 1967 figure. There were established offices, Technicentres and distribution centres in 23 countries around the world. Revenues for 1976 were in excess of $225 M. Based in 23 countries, Technicon employees numbered over 4000. Of course Technicon's fortunes have continued since the mid 1970s, and there are now over 40 instrument systems and hundreds of after-market items.

This survey is not intended to be an exhaustive account of Technicon's business operations, but it illustrates a company that has tackled multi-dimensional problems with experts from many disciplines. Many recent products have been designed to be operated virtually anywhere by non-specialist personnel. The company provide a multidimensional service in their instrument systems, support back-up, customer training, system servicing, consumables supply, reagents and standards, various channels for dissemination of information (technical brochures, demonstrations etc. [66]), research and development investment, and task teams.

The link between science and technology (invention and its application) has, in the case of the AutoAnalyzer, been more than a tenuous one. As well as the many experts from a multitude of disciplines becoming involved in this new innovation of analytical science, also theoretical science 'got its hand dirty'. The company do not appear to advertise on any regular basis in the normal journals—perhaps this is a measure of their success. (The author managed to dig one out from a journal of the late 1950s.) In this resumé of both inventor and the Technicon Company, certain distinct features have been highlighted which have all been contributors to technical, scientific and business success. Should any of these key elements have been omitted, it may even be conjectured that set-ups like the AutoAnalyzer, for instance, would perhaps, in later years, have seemed merely a good idea at the time! It has been the link between pure and applied science and industry which ultimately furnished a success story for the AutoAnalyzer. In many British industries such links are often insubstantial. In the years following the end of the Second World War, in Britain in particular—when industrial secrecy abounded—many instrument companies failed to realize much in the way of technical progress. They perhaps had 'me-too' products. The Brain Drain [75] did not help the situation: what appeared to be a healthy instrument business in the UK slowly drifted abroad [76].

There are many factors which have contributed to Britain's relatively poor performance in the area of the instrument business—particularly when contrasted against that in the USA—but perhaps the overriding constraint preventing a coherent growth of the industry has been a lack of enthusiasm about analytical science. The business is extant with multivarieties of systems from various parts of the globe, and now many are computer interfaced. The British industry is now trying to make a come-back and perhaps it should take some lessons from those learnt so hard, but well, by Technicon and the Skeggs AutoAnalyzer.

There are many examples in the literature of Britain failing to capitalize on discovery and where many of her own inventions have ended up elsewhere. The idea that invention often comes about at the interface of between two or more disciplines was recognized in the 1930s with the discovery of polythene as M. W. Perrin, of the Wellcome Foundation Ltd, pointed out in 1953:

> Since a chance discovery may, and probably will, lie outside the field of immediate interest to those who make it, rapid and effective recognition is more likely if the research man concerned has as wide a knowledge as possible of science and its application in industry. The contribution of the specialist is certainly needed and this, again, is clearly shown up in the polythene story but it also serves to emphasize the importance of collaboration in, and appreciation of, the work of others in widely different fields of academic and applied research [77].

University departments have been established in more recent years to tackle such problems with respect to the instrument business in Britain. This new awareness can only serve to accentuate the fundamental importance of analytical science in industry.

### References

1. Skeggs, Jr, L. T., *The American Journal of Clinical Pathology*, **28** (1957), 311.
2. Henahan, J. F., *Chemical and Engineering News*, **48** (1970), 55.
3. Skeggs, Jr, L. T., *Advances in Automated Analysis*, **1** (New York, 1970), 3.
4. Bernhart, F. W. and Skeggs, L. T., *Journal of Biological Chemistry*, **147** (1943), 19.
5. Skeggs, Jr, L. T., and Leonards, J. R., *Proceedings of the Society for Experimental Biological Medicine*, **63** (1946), 63.
6. Skeggs, Jr, L. T., Leonards, J. R. and Myers, V. C., *Federal Proceedings*, **7** (1948).

7. Encyclopaedia Britannica, *World Who's Who in Science* (1973).
8. *World Who's Who in Science.*
9. Abel, J. J., Rowntree, L. G. and Turner, B. B., *Journal of Pharmacology and Experimental Therapy*, **5** (1914), 275.
10. Guarino, J. R. and Guarino, L. J., *Science*, **115** (1952), 285.
11. Stanley, R., *The Introduction of Automated Analysis in the Chemical and Allied Industries in Britain* (University of Manchester Institute of Science and Technology, 1982), 398. (Hereafter cited as *Automated Analysis in Britain.*)
12. Skeggs, Jr, L. T. and Heisler, C. R., *Proceedings of the Society for Experimental Biological Medicine*, **72** (1949), 540.
13. Skeggs, Jr, L. T. and Leonards, J. R., *Science*, **108**, 212–213.
14. Henahan, op. cit., 55.
15. Skeggs, Jr, L. T., *Analytical Chemistry*, **38** (1966), 32A.
16. *Automated Analysis in Britain*, 411.
17. *The Technicon Story* (an unpublished account of the history of the company), 13.
18. Ibid., 14.
19. US Patent 2,797,149 (1957).
20. Ibid., 12, 17–19.
21. *Riding Along*, 79–86.
22. Interview: Whitehead, E. C. (1980).
23. *Analytical Chemistry*, **52** (1980), 633A.
24. Skeggs, Jr, L. T., *The American Journal of Clinical Pathology*, **28** (1957), 311.
25. Ferrari, A., *Proceedings of the New York State Association of Public Health Laboratories*, **42** (1962), 36.
26. Skeggs, Jr, L. T. and Hochstrasser, H., *Clinical Chemistry*, **10** (1964), 918.
27. *Automated Analysis in Britain*, 487.
28. Ibid., 797.
29. *Riding Along.*
30. Henahan, op. cit., 58.
31. Skeggs, Jr, L. T., Kahn, J. R., Levine, M., Dorer, F. and Lentz, K. E., *Circulation Research*, **37** (1975), 715–724.
32. Skeggs, Jr, L. T., Kahn, J. R., Levine, M., Dorer, F. and Lentz, K. E., *Circulation Research*, **39** (1976), 400.
33. Skeggs, Jr, L. T., Dorer, F. E., Kahn, J. R., Lentz, K. E. and Levine. M., *The American Journal of Medicine*, **60** (1976), 737.
34. *Automated Analysis in Britain*, 788.
35. Personal communication and interview, Varley, H. (formerly of the Department of Biochemistry, Royal Infirmary, Manchester), (1979 and 1980); Varley. H., *Proceedings of The Association of Clinical Biochemists*, **4** (1966), 95.
36. Stanley, R., *Laboratory Practice*, **29** (1980), 1179; Sanders, P. G. Development of CFA (lecture: Summer School of Automatic Chemical Analysis, 1980); Sanders, P. G., *British Journal of Hospital Equipment* (May 1975), 5.
37. Sanders, ibid., 5; Anderson, A. G., *St. Bartholomew's Journal* (November 1958), 311; Flynn, F. V., *Proceedings of the Association of Clinical Biochemists*, **3** (1965), 272; Varley, op. cit., 93; Whitehead, T. P., *Annals of Clinical Biochemistry*, **8** (1971), 1; Varley, H., *Annals of Clinical Biochemistry*, **11** (1974), 161.
38. Stanley, R., *Laboratory Practice*, **29** (1980), 1179.
39. *Automated Analysis in Britain*, 492.
40. *Technicon Corporation Annual Report* (1969), 2.
41. *Automated Analysis in Britain*, 498.
42. *World's Who's Who in Science.*
43. *The Technicon Story*, op. cit., 7.
44. The Technicon Company technical brochure 10–49 5M E, *Lab-Aid:* Laboratory Filing System, personal communication (1980).
45. Cunningham, R. M. and Roberts, W. C., *Advances in Automated Analysis*, **1** (New York 1970), 367.
46. Europa, D. L., *Advances in Automated Analysis*, 373.
47. Mayfield, D. W., *Advances in Automated Analysis*, 377.
47. Mayfield, D. W., *Advances in Automated Analysis*, **1** (New York 1970), 377.
48. *Automated Analysis in Britain*, 530.
49. Stein, W. H. and Moore, S., *Journal of Biological Chemistry*, **176** (1948), 337; Moore, S. and Stein, W. H., *Science*, **180** (1973), 459; Moore, S., *Chemistry and Biology of Peptides* (Michigan, 1972), 631.
50. Martin, A. J. P. and Synge, R. L. M., *Biochemical Journal*, **35** (1941), 1358; Martin, A. J. P., *Annual Review of Biochemistry*, **19** (1950), 517; Synge, R. L. M., *Analyst*, **71** (1946), 256; Moore, S. and Stein, W. H., *Science*, **180** (1973), 459.
51. *Automated Analysis in Britain*, 256 and 515.
52. *The Technicon Story*, 15.
53. *Automated Analysis in Britain*, 797.
54. Ibid., 520.
55. Ibid., 516–529.
56. *Technicon Corporation Annual Report* (1969), 28.
57. *Automated Analysis in Britain*, 535.
58. *Technicon Corporation Annual Report* (1971).
59. *Advances in Automated Analysis*, 465.
60. *Automated Analysis in Britain*, 541.
61. Ibid., 546.
62. Personal communication, Scholes, P. H. (1979 and 1981).
63. *Technicon Corporation Annual Report* (1975).
64. *The Technicon Story*, 28.
65. Stanley, R., *Laboratory Practice*, **29** (1980), 1179.
66. *Technicon AutoAnalyzer Bibliography 1957/1967; Technicon Bibliography 1967–1973; Technicon Bibliography Supplement 1, 1974; Technicon Bibliography Supplement 2, 1975; Technicon Bibliography Supplement 3, 1975; Technicon Bibliography* Papers Nos. 2540–2702 and 8026–9200, n.d.
67. *Advances in Automated Analysis*, 3.
68. *Advances in Automated Analysis*, v, xiii and 288.
69. Smythe, W. J., Shamos, M. H., Morgenstern, S. and Skeggs, Jr, L. T., *Automation in Analytical Chemistry*, **1** (New York, 1968), 105.
70. Bryan, D. J., Wearne, J. L., Viau, A., Musser, A. W., Schoonmaker, F. W. and Thiers, R. E., *Automation in Analytical Chemistry* (New York, 1966), 423.
71. Finley, P. R., Gillott, L., Peterson, D., Anderson, F. and Boxman, E., *Advances in Automated Analysis*, 145.
72. Isreeli, J. and Smythe, W., *Advances in Automated Analysis*, 13.
73. *Technicon Corporation Annual Report* (1972).
74. *Automated Analysis in Britain* 489.
75. *The Brain Drain*, Cmnd 3417, (1967).
76. *Automated Analysis in Britain*, 376.
77. Perrin, M. W., *Research* (1953), 111.

### Author's note

The importance of a multidisciplinary approach to instrument development, marketing and support has been illustrated with the early history of individuals who came together to build a company, which eventually gave rise to the clinical laboratory instrument industry as we know it today.

The illustration stops in 1976, although the company's history does not. This year Technicon celebrates its 45th anniversary. In 1978 Technicon opened its Chemical and Biological Manufacturing Operation at Middletown, Virginia, one of the most modern reagent manufacturing facilities that exist today.

In 1980 the haematology product line was expanded and updated with the Technicon H6000 combined blood count and leukocyte differential counter.

Also in 1980 Technicon became part of the Revlon Health Care Group. Revlon, publicly known for its cosmetics, entered the health-care field in 1966 and encompasses pharmaceuticals, contact lens and optical companies, proprietary drugs and medications, a clinical laboratory chaain, plasma and blood product companies. With the acquisition of Technicon Corporation, Revlon broadened its position in diagnostics and opened entirely new avenues of opportunity.

Technology developments continued, and in 1981 the Technicon RA-1000 system was introduced, featuring a new hydraulic principle reducing to negligible proportions the contamination and carry-over previously experienced on all types of instrument.

In 1982 the New York Chapter of the American Association for Clinical Chemistries awarded the annual Van Slyke Memorial to Edwin C. Whitehead for his services to laboratory medicine. This year Edwin C. Whitehead created the Whitehead Foundation at the Massachussetts Institute of Technology with a pledge of \$135 M for the advancement of biomedical research.

The first Technicon AutoAnalyser was presented to the Medical Sciences Division of the National Museum of American History of the Smithsonian Institution, Washington, D.C.

